# Diffuse idiopathic pulmonary neuroendocrine cell hyperplasia diagnosed by transbronchial lung cryobiopsy: a case report

**DOI:** 10.1186/s13256-017-1254-y

**Published:** 2017-04-07

**Authors:** R. Sauer, S. Griff, A. Blau, A. Franke, T. Mairinger, C. Grah

**Affiliations:** 1 Institute of Pathology, HELIOS Klinikum Emil von Behring, Walterhöferstr. 11, Berlin, 14165 Germany; 2 Department of Respiratory Medicine, Gemeinschaftskrankenhaus Havelhöhe, Berlin, Germany; 3 Group Practice of Respiratory Medicine, Klosterstraße 34/35, Berlin, Germany

**Keywords:** Case report, DIPNECH, Obliterative bronchiolitis, Cryobiopsy

## Abstract

**Background:**

Micronodular lesions are common findings in lung imaging. As an important differential diagnosis, we describe a case of diffuse idiopathic pulmonary neuroendocrine cell hyperplasia; it is notable that the diagnosis of diffuse idiopathic pulmonary neuroendocrine cell hyperplasia is often delayed. This case provides supporting evidence to establish lung biopsy by cryotechnique as the option of first choice when considering a diagnostic strategy for micronodular lung lesions.

**Case presentation:**

We report a case of a 65-year-old white woman who presented with obstructive symptoms of chronic coughing and dyspnea confirmed by conventional lung function tests. A computed tomography scan presented disseminated micronodules in all the lobes of her lungs. With the help of bronchoscopic cryobiopsy it was possible to obtain a high yield sample of lung parenchyma. On histologic examination, the micronodules correlated with a diffuse neuroendocrine cell hyperplasia. In the context of clinical symptoms, radiological aspects, and histomorphological aspects we made the diagnosis of a diffuse idiopathic pulmonary neuroendocrine cell hyperplasia. Obstructive symptoms were treated with inhaled steroids and beta-2-mimetics continuously. A comparison between current computed tomography scans of our patient and scans of 2014 revealed no significant changes. Last ambulatory checks occurred in January and May of 2016. The course of disease and the extent of limitation of lung function have remained stable.

**Conclusions:**

The diagnosis of diffuse idiopathic pulmonary neuroendocrine cell hyperplasia is best made in a multidisciplinary review including clinical presentation, lung imaging, and histomorphological aspects. This report and current literature indicate that transbronchial lung cryobiopsy can be used as a safe and practicable tool to obtain high quality biopsies of lung parenchyma in order to diagnose micronodular lesions of the lung.

## Background

Diffuse idiopathic pulmonary neuroendocrine cell hyperplasia (DIPNECH) is a rare idiopathic disease, which was named by Aguayo *et al*.; it is associated with neuroendocrine cell hyperplasia and obliterative bronchiolitis [1]. Patients are typically older women and non-tobacco smokers who are affected by obstructive symptoms such as coughing and dyspnea [2]. On histologic examination, DIPNECH presents as a scattered nodular or linear proliferation of neuroendocrine cells either superficial to the basement membrane of bronchial or bronchiolar epithelium or in the form of tumorlets beyond it. Tumorlets are neuroendocrine cell proliferates smaller than 5 mm in diameter. The World Health Organization defines DIPNECH as a praeneoplastic condition. Patients may develop synchronous or subsequent carcinoid tumors, which measure more than 5 mm whereas tumorlets measure less than 5 mm [3, 4]. DIPNECH is known to have a good prognosis. Most cases show stability in symptoms and radiological findings. Therefore a watch and wait strategy seems the method of choice. However, a few patients show rapid clinical progression [2]. In cases of progressive disease a surgical excision of dominant lesions and somatostatin analogs may be considered a therapy option [5]. Similar to other obstructive lung diseases, complications such as acute exacerbation including bronchitis and pneumonia can occur. A standard procedure in diagnostic strategy and therapy of DIPNECH has not yet been established.

## Case presentation

In November 2014, a 65-year-old white woman with a history of progressive dyspnea presented to our hospital for evaluation. She complained about dyspnea on exertion and had had several infectious exacerbations of chronic obstructive pulmonary disease (COPD) during the last year, one requiring hospitalization. During those exacerbations she had been treated with tapering doses of systemic corticosteroids showing improvement in hypoxemia and obstructive symptoms. At the point of presentation her medication for inhalation included: budesonide/formoterol 400 μg/12 μg twice a day, tiotropium bromide 18 μg once a day, and salbutamol as needed, which she was using at least once a day. Her general patient history was negative for atopic diseases or allergies. At the age of 54 she was diagnosed as having COPD due to dyspnea and typical lung function tests. She stated that she had never smoked tobacco. A skin prick test revealed no hypersensitivity. Since the diagnosis of COPD had been established she was regularly followed-up by a pulmonologist. At hospital admission, her general condition was slightly disturbed. She was hypoxic at rest. The saturation level of oxygen in hemoglobin (SaO_2_) was 92%, without providing indication for oxygen therapy.

**Fig. 1:**
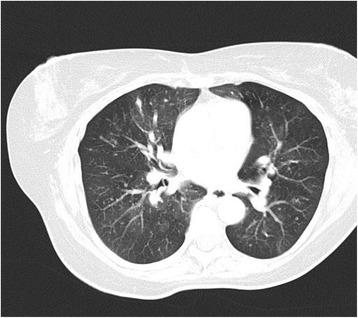
Axial image from chest computed tomography scan showing multiple scattered pulmonary nodules and mosaicism

A body plethysmography showed forced vital capacity (FVC) 1.47 L (62%), forced expiratory volume in 1 second (FEV_1_) of 0.8 L (40%), airway resistance of 1.62 kPa × second/L (538.9%), residual volume of 3.30 L (188%), and a total lung capacity of 4.50 L (108%). Those results were evaluated as severe obstruction with massive air trapping compatible with the diagnosis of COPD. During the further evaluation process different methods of imaging were conducted. A chest X-ray showed well-ventilated lungs and discreet apical pleural callosity. Transthoracic echocardiography showed normal cardiac structures without evidence of pulmonary hypertension. In a computed tomography (CT) scan, disseminated small nodules between 2 and 4 mm in all lobes, ground glass characteristic, and partial mosaicism on both lungs were strikingly apparent. Focal bronchial thickening could be seen. Furthermore, mediastinal lymph nodes were heightened (Fig. 1).

As a result of lung imaging, bronchoscopy was initiated. Forceps biopsies of her central airways were taken, showing normal respiratory mucosa without any changes suspicious for a specific inflammation. In particular, granulomas or giant cells could not be detected. Due to ongoing clinical suspicion for interstitial lung disease a further attempt of forcing a histological diagnosis was made.

**Fig. 2:**
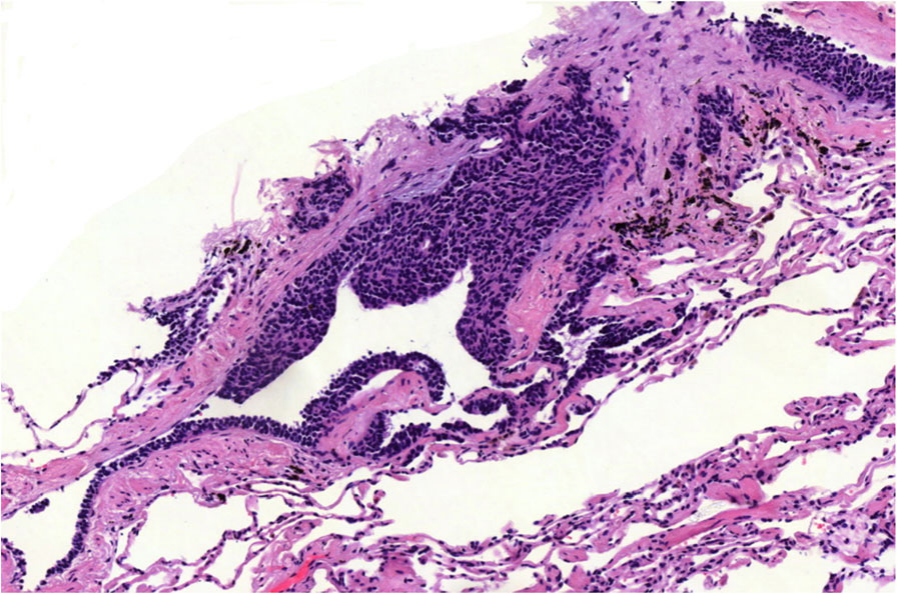
Hematoxylin and eosin stained slide shows bronchiole with linear and nodular proliferates of neuroendocrine cells bulging into the lumen

**Fig. 3:**
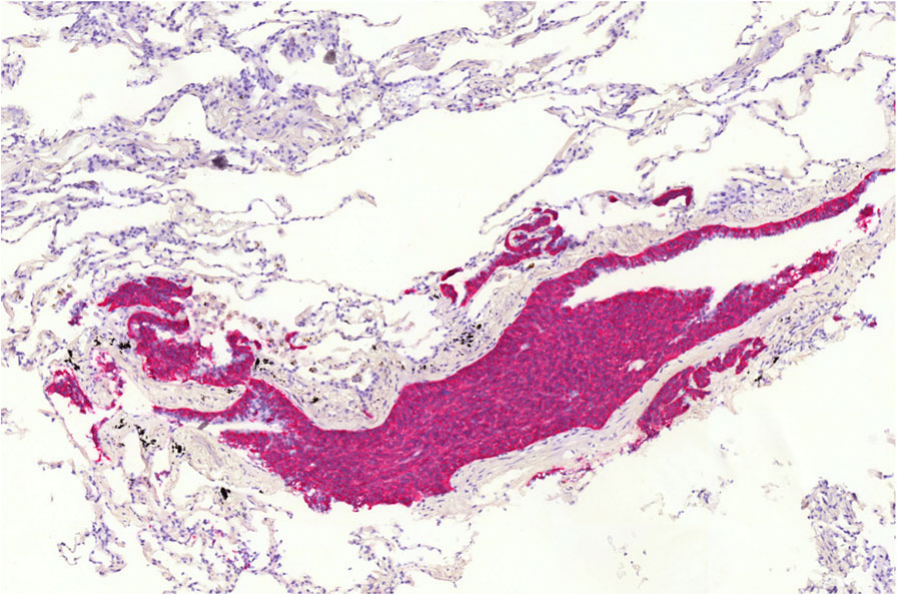
Strong positivity in immunohistochemical analysis with synaptophysin

By method of transbronchial cryobiopsy (ERBECRYO2, diameter 1.9 mm; Tübingen, Germany), lung tissue samples of the middle and inferior lobes of her right lung were gained. The material was formalin fixed and paraffin embedded, cut into 4 μm-thick sections and stained with hematoxylin and eosin (H&E). The fragmented biopsy measured an overall area of 30.9 mm^2^ compared with the initial biopsy of 7.5 mm^2^ (Mirax Viewer Image Software Version 1.12, Zeiss Microimaging, Oberkochen, Germany and 3D Tech, Budapest, Hungary). Besides small airways, a regular lung parenchyma with an alveolar basic structure could be seen. The bronchioles were lined by a regular respiratory epithelium. Furthermore, in her bronchiolic mucosa, linear and nodular proliferates of small uniform cells with round to slightly ovoid nuclei and disperse chromatin were located within the epithelial basement membrane and bulged into the lumina (Fig. 2). Peribronchiolar and perivascular aggregates of cells showing this morphology were also found, measuring less than 5 mm in diameter at maximum. No signs of malignancy could be found; neither could we find any mitotic activity, desmoplastic stroma reaction, or any invasive aspect. In association with the described cell cluster, slight fibrosis could be seen. In the Elastica van Gieson stain, the walls of some bronchioles were broadened and contained an increased amount of elastic fibers. Immunostainings revealed strong positivity for synaptophysin (Fig. 3), chromogranin A, and CK7 in the cell cluster described. The proliferative index determined by Ki67% (Mib-1) was beneath 1% (antibodies by Roche, Rotkreuz, Switzerland). Based on these findings, the diagnosis of a neuroendocrine cell hyperplasia could be made and a possible association with DIPNECH was noted.

Due to concomitant bronchitis our patient received antibiotic therapy. Obstructive symptoms were treated with inhaled steroids and beta-2-mimetics continuously. Comparing current CT scans of our patient with scans in 2014 no significant changes are described. Last ambulatory checks occurred in January and May of 2016. The course of disease and the extent of limitation of lung function have remained stable.

## Discussion

On the background of clinical presentation and lung imaging the microscopic morphology is compatible with a manifestation of DIPNECH. Our patient presented has gone through a history of obstructive lung disease including symptoms like exertional dyspnea with slight relief under anti-obstructive therapy. Also, lung function tests have repeatedly shown severe obstruction. In comparison to a typical patient who has COPD, our patient does not have a history of tobacco smoking. In the past and recent literature, DIPNECH has been described to be potentially associated with obliterative bronchiolitis and respectively could be the cause of chronic obstruction [1, 2, 6, 7].

On histologic examination, the wall of her bronchioles were thickened and showed a linear and nodular neuroendocrine cell hyperplasia, in which an incomplete obliteration of these airways could be shown by cell bulging into the airway lumen. However, the complete morphological picture of an obstructive bronchiolitis is not comprised in the obtained biopsy. However, it can easily be imagined that even an incomplete obliteration of bronchioles can cause obstruction comparable to symptoms induced by a follicular bronchiolitis, in which prominent lymphatic tissue obliterates bronchiolic lumina. Furthermore, broncho-obstruction can be caused or at least aggravated by different vasomodulating and bronchomodulating peptides produced in hyperplastic/neoplastic pulmonary neuroendocrine cells [8]. Any of these changes may have caused the obstructive symptoms of this patient, including typical lung function tests, potentially leading to earlier diagnoses of chronic obstructive lung disease or intrinsic asthma. In this regard, it is notable that the final diagnosis of DIPNECH is often delayed [7].

Micronodular lesions of the lung can be coincidental diagnostic findings in lung imaging. Also, DIPNECH is sometimes asymptomatic and may be diagnosed accidentally [2]. To consider different courses of disease the term “DIPNECH syndrome” has been proposed [9]. In CT imaging DIPNECH can present not only with multiple small nodules in both lungs but also with bronchial wall thickening and mosaic perfusion [10]. Rare radiological findings include fibrotic changes comparable to a restrictive pattern in usual interstitial pneumonia [11]. In addition to lung imaging, surgical lung biopsies are usually required to diagnose DIPNECH and corresponding obliterative bronchiolitis [6]. Any other symptomatic or asymptomatic micronodular lesion, for example sarcoidosis, usually requires an open lung biopsy to be clarified as well. Further diagnostic tools like an octreotide scan and positron emission tomography (PET)-CT are also helpful. Blood examinations might reveal elevated serum levels of chromogranin A [5].

In this special case, transbronchial cryobiopsy was used to obtain a good quality biopsy of lung parenchyma from which it was possible to generate the diagnosis of DIPNECH. This method has an advantage over the use of conventional transbronchial forceps because alveolar tissue is contained more often and shows fewer crush artefacts [12, 13]. Besides, the cryotechnique method seems safer and less straining for patients compared to open biopsy methods [14]. The risk of significant bleeding in transbronchial cryobiopsy does not differ from that of using conventional forceps [15]. In particular, concerning histomorphological analysis of interstitial lung diseases, the cryotechnique can be used in combination with flexible catheters to reach peripheral areas of the lung [16]. Transbronchial cryobiopsy has a particularly good diagnostic yield for diseases diffusely involving the lung parenchyma [17, 18]. In summary, transbronchial cryobiopsy can be regarded as an ideal method for diagnosing DIPNECH.

## Conclusions

The diagnosis of DIPNECH is best made in a multidisciplinary review including clinical presentation, lung imaging, and histomorphological aspects. Current literature indicates that transbronchial lung cryobiopsy can be used as a safe and practicable tool to obtain high quality biopsies of lung parenchyma in order to diagnose micronodular lesions of the lung. Therefore, this case provides supporting evidence to establish lung biopsy by cryotechnique as the option of first choice before using surgical biopsy techniques.

## Abbreviations

COPD: Chronic obstructive pulmonary disease
CT: Computed tomography
DIPNECH: Diffuse idiopathic pulmonary neuroendocrine cell hyperplasia
FEV_1_: Forced expiratory volume in 1 second
FVC: Forced vital capacity
H&E: Hematoxylin and eosin
PET: Positron emission tomography
SaO_2_: Saturation level of oxygen in hemoglobin
